# *In vitro* Magnetic Stimulation: A Simple Stimulation Device to Deliver Defined Low Intensity Electromagnetic Fields

**DOI:** 10.3389/fncir.2016.00085

**Published:** 2016-11-03

**Authors:** Stephanie Grehl, David Martina, Catherine Goyenvalle, Zhi-De Deng, Jennifer Rodger, Rachel M. Sherrard

**Affiliations:** ^1^Sorbonne Universités, UPMC Univ Paris 06 & CNRS, IBPS-B2A, UMR 8256 Biological Adaptation and AgeingParis, France; ^2^Experimental and Regenerative Neuroscience, School of Animal Biology, the University of Western Australia, PerthWA, Australia; ^3^Institut Langevin, ESPCI ParisTech & CNRS, UMR7587 INSERM ERL U979Paris, France; ^4^Non-invasive Neuromodulation Unit, Experimental Therapeutics and Pathophysiology Branch, Intramural Research Program, National Institute of Mental Health, National Institutes of Health, BethesdaMD, USA; ^5^Department of Psychiatry and Behavioral Sciences, Duke University School of Medicine, DurhamNC, USA

**Keywords:** magnetic stimulation, low intensity repetitive magnetic stimulation, rTMS, LI-rMS, computational modeling, electric field, magnetic field, magnetic coil design

## Abstract

Non-invasive brain stimulation (NIBS) by electromagnetic fields appears to benefit human neurological and psychiatric conditions, although the optimal stimulation parameters and underlying mechanisms remain unclear. Although, *in vitro* studies have begun to elucidate cellular mechanisms, stimulation is delivered by a range of coils (from commercially available human stimulation coils to laboratory-built circuits) so that the electromagnetic fields induced within the tissue to produce the reported effects are ill-defined. Here, we develop a simple *in vitro* stimulation device with plug-and-play features that allow delivery of a range of stimulation parameters. We chose to test low intensity repetitive magnetic stimulation (LI-rMS) delivered at three frequencies to hindbrain explant cultures containing the olivocerebellar pathway. We used computational modeling to define the parameters of a stimulation circuit and coil that deliver a unidirectional homogeneous magnetic field of known intensity and direction, and therefore a predictable electric field, to the target. We built the coil to be compatible with culture requirements: stimulation within an incubator; a flat surface allowing consistent position and magnetic field direction; location outside the culture plate to maintain sterility and no heating or vibration. Measurements at the explant confirmed the induced magnetic field was homogenous and matched the simulation results. To validate our system we investigated biological effects following LI-rMS at 1 Hz, 10 Hz and biomimetic high frequency, which we have previously shown induces neural circuit reorganization. We found that gene expression was modified by LI-rMS in a frequency-related manner. Four hours after a single 10-min stimulation session, the number of c-fos positive cells increased, indicating that our stimulation activated the tissue. Also, after 14 days of LI-rMS, the expression of genes normally present in the tissue was differentially modified according to the stimulation delivered. Thus we describe a simple magnetic stimulation device that delivers defined stimulation parameters to different neural systems *in vitro*. Such devices are essential to further understanding of the fundamental effects of magnetic stimulation on biological tissue and optimize therapeutic application of human NIBS.

## Introduction

Non-invasive brain stimulation (NIBS), using electric or magnetic fields, is increasingly used in neurological and psychiatric treatment (Pascual-Leone, 2006; Pell et al., 2011). However, clinical outcomes of extrinsic brain stimulation are variable (Wassermann and Zimmermann, 2012), revealing how little we know about the cellular mechanisms underlying the effects of different protocols. Even within the domain of magnetic stimulation there are different approaches. Classical transcranial magnetic stimulation (TMS) or repetitive TMS (rTMS) delivers focal high intensity stimulation (peak magnetic field of 1–2 T, generating electric fields, *E*≥100 V/m) to depolarise underlying neurons and modulate specific neural circuits. Newer low- or pulsed-field magnetic stimulation (LFMS, PMF) delivers diffuse low-intensity stimuli (μT-mT range, *E* ≤ 1 V/m) that are also biologically effective: modifying cortical function (Capone et al., 2009; Robertson et al., 2010), brain oscillations (Cook et al., 2004; Modolo et al., 2013) and metabolism (Volkow et al., 2010), as well as neurological dysfunction (Martiny et al., 2010; Rohan et al., 2014). We have combined these two approaches creating a small rodent coil to deliver focal low-intensity magnetic stimulation (LI-rTMS), and found that 2 weeks of LI-rTMS can reorganize neural circuits (Rodger et al., 2012; Makowiecki et al., 2014). However, although LFMS now forms an additional tool for NIBS therapies (Shafi et al., 2014), the mechanisms underlying the effects of low intensity magnetic stimulation remain ill-defined.

To better understand the effects of magnetic stimulation on neural tissue, it is necessary to study defined magnetic, and therefore electric, fields in simple systems wherein the experimental variables can be precisely controlled, e.g., in *in vitro* models. However, a significant challenge to such investigations is the delivery of defined repetitive magnetic stimulation (rMS) conditions to the culture dish (Basham et al., 2009). Some rMS studies have used high intensity stimulation through human coils (Post et al., 1999; Rotem and Moses, 2008; Stock et al., 2012; Vlachos et al., 2012; Ma et al., 2013; Lenz et al., 2016) that can only be applied outside the incubator, changing the tissue environment and therefore its response to magnetic fields (RamRakhyani et al., 2013). In addition, the efficiency of electric field induction depends on the relative sizes of coil and target (Weissman et al., 1992; Deng et al., 2013), thus the electric fields induced by human coils in small *in vitro* targets are different to those generated in the human brain, so that information obtained cannot be directly translated back to the clinic. In low-intensity stimulation (LI-rMS) studies, solenoids (Di Loreto et al., 2009; Varro et al., 2009) or coils made “in house” have been applied to one-off experiments on cultured neurons/slices (Ahmed and Wieraszko, 2009; Rotem et al., 2014) or isolated nerves (Maccabee et al., 1993; Basham et al., 2009; RamRakhyani et al., 2013; Ahmed and Wieraszko, 2015) that do not permit on-going stimulation sessions to model treatment-based protocols. Moreover, given that NIBS acts on complex neural circuits, stimulation parameters should ideally be assessed in culture models which retain some neural circuitry: e.g., organotypic hippocampal (Hausmann et al., 2001; Hogan and Wieraszko, 2004; Vlachos et al., 2012; Lenz et al., 2016) and cortico-striatal slices, hindbrain explants (Chedotal et al., 1997; Letellier et al., 2009) or microfluidic circuit cultures (Szelechowski et al., 2014). However, these diverse culture systems have unique dimensions and culture conditions, highlighting the need to establish a reliable and flexible LFMS/LI-rMS system that can be tailored to deliver a defined magnetic field that induces an electric field of predicted intensity and direction at a particular location within each culture.

The effects of magnetic stimulation are thought to be due to the electric field and current induced within the target tissue (Pell et al., 2011; Di Lazzaro et al., 2013). Although, a few studies have profited from indwelling electrodes to measure TMS-induced electric fields in the brain (macaque, Lisanby et al., 2001; human, Wagner et al., 2004), the currents actually generated will vary according to the cellular components involved, specifically cell membrane resistance and capacitance, which in turn vary with the presence/absence of myelin and/or the distribution of ion channels (Chan and Nicholson, 1986; Wagner et al., 2014). However, in human patients/subjects without such electrodes, modeling or calculation is needed to define the electric field (Deng et al., 2013; Lu and Ueno, 2013; Janssen et al., 2015). We aimed to create a symmetrical, unidirectional homogenous magnetic field perpendicular to our culture tissue. Thus the electromagnetic field of any stimulation protocol determined to have important biological effect can be reproduced in a larger human head using appropriately designed large coils.

Here, we describe the design and construction of a generic stimulation device suitable for long-term LI-rMS *in vitro* that can be adapted to specific culture conditions. Specifically, we have built a device that applies LI-rMS at a range of frequencies to hindbrain explants containing a model of axonal injury and repair, the lesioned olivocerebellar path (Chedotal et al., 1997; Letellier et al., 2009). We use stimulation at an intensity that alters intracellular calcium and gene expression (Mattsson and Simkó, 2012; Grehl et al., 2015), provides neuroprotection (Yang et al., 2012) and reorganizes neural circuits *in vivo* (Rodger et al., 2012; Makowiecki et al., 2014). The wide range of parameters controlled by our fully automated magnetic stimulator and coil system will facilitate comparison of different stimulation protocols in a range of *in vitro* models, contributing to the optimisation of focal low-intensity NIBS application to the clinic.

## Methods and Results: Stimulation Device Design, Construction and Validation

Using *in vitro* tissue to research the effects of magnetic stimulation has the advantage of precise control and isolation of experimental variables, increasing standardization and reproducibility of results and the possibility of comparison between studies. This study created a magnetic stimulation device whose design was compatible with long-term stimulation protocols within an incubator, simultaneously delivering defined electromagnetic fields to tissue in multiple culture wells, without either eddy-current cross-interference or disturbing the culture environment. Moreover, the device is automatic with adjustable parameters for pulse waveform, frequency and field intensity, in order to be applicable to different culture settings. Parameters identified as one possible solution for our specific experimental requirements are given in **Table 1**.

**Table 1:** Parameters chosen for this specific *in vitro* set-up were identifed using a combination of several software programs.

*Restricted parameters*	Outer	Rise-time	I
	30 mm	100 μs	2 A			

***Chosen parameters***	**Inner Ø**	**Height**	**No. Turns**	**Copper Wire** Ø	**L**	**R**
	10 mm	3.5 mm	119	0.40 mm	224 μH	11.6 Ω

*Ø = diameter, I = electronic circuit current, L = inductance, R = Resistance.*

### Requirements for *In vitro* Magnetic Stimulation

*In vitro* culture, whether of isolated neurons and glia or neurons within intact circuits of three-dimensional organotypic explants, has physical restraints that have to be accommodated during magnetic stimulation protocols: sterility, stable temperature of the incubator and constant gas atmosphere (95% air plus 5% CO_2_) for maintaining pH. Thus cultures have to be stimulated within the restricted incubator environment, with the coils being outside a closed culture dish. Moreover, in contrast to using large Helmholtz solenoids, small coils for individual culture wells maximize throughput of different stimulation parameters.

**FIGURE 1:**
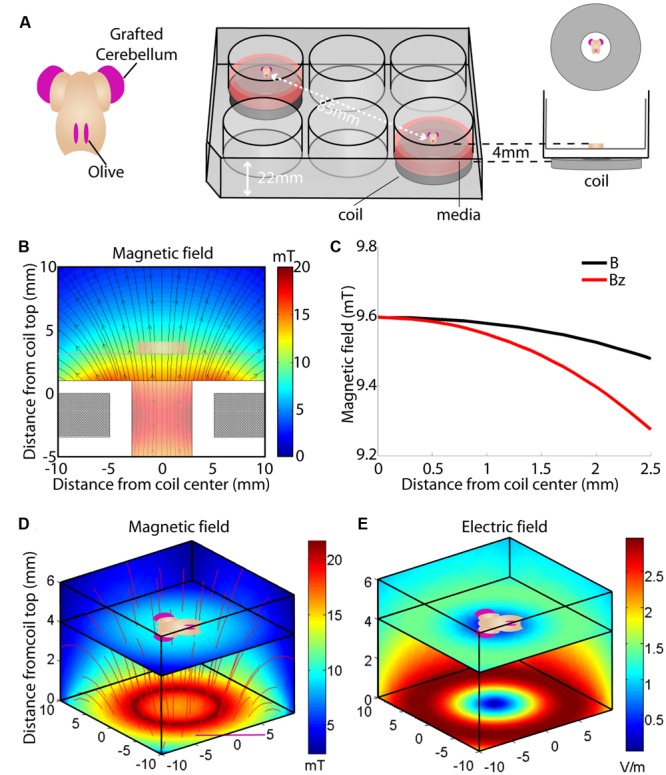
**Overview of the culture set-up **(A)** and induced magnetic **(B–D)** and electric field **(E)**. (A)** Hindbrain explants are dissected so that they contain a central brainstem containing the inferior olive caudally and the 2 hemicerebellar plates at each side (beige). For our lesion model we graft two denervated hemicerebellae (cerise pink) adjacent to an intact explant (left panel). Explants are cultured on Millicell membranes in six-well plates (middle panel). The organotypic hindbrain samples (beige) were cultured on a membrane ∼4 mm above the top of the coil, placed underneath the culture well (right panel). **(B)** Modeled overview of the coil and its generated magnetic field. Gray squares at the bottom of the image show a cross-section of the coil wiring (see **Table 1** for coil components and dimensions). White surround corresponds to the coil’s plastic shell. Colors correspond to the magnetic field strength and black arrows to magnetic field direction 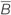. The beige block in the center of the image shows the location of the explant on a horizontal plane 4 mm above the coil. **(C)** Schematic overview of the different magnetic field components at 4 mm distance from the base of the coil. The magnetic field 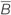 is almost exclusively comprised of a vertical field component B_z_ (red line) up to 2.5 mm from the coil axis, whereas thehorizontal component is effectively zero within this region. **(D)** Modeled overview of magnetic field strength (mT; colors) and direction 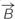 (red lines) starting at the top of the coil wiring (horizontal plane at 0 mm). The explant lies 4 mm above within the unidirectional magnetic field. **(E)** Modeled overview of the induced electric field from the top of the coil wiring (Horizontal plane at 0 mm). Colors indicate the electric field strength (V/m). The explant at 4 mm shows that the areas of interest (cerise pink; grafted hemicerebellae and inferior olive) lie in the same electric field.

Our particular *in vitro* experiments required long-term culture and stimulation of multi-axial (L × W × H, 5 × 5 × 1 mm) organotypic mouse hindbrain explants cultured on 30 mm Millipore membranes (Millipore, USA) in six-well culture plates [Techno Plastic Products (TPP), Switzerland] and incubated at 35°C in 95% air plus 5% CO_2_ (Chedotal et al., 1997; Letellier et al., 2009). Details of dissection and culture procedures are provided below (Biological Validation). Since explants on insert membranes are closer to the bottom of the culture plate than the top, to minimize coil-tissue distance, the coils were located directly underneath the culture wells. Distance from the top of the coil to the multi-axial target tissue was 3.5–4.5 mm (1 mm tissue depth; **Figure 1A**).

### Requirements for Magnetic and Induced Electric Field at the Target Tissue

A time varying primary current in a coil creates a time varying magnetic field. The Maxwell–Faraday equation

(1) $$\begin{array}{c} -\frac{d}{dt}\iint \vec{B}.d\vec{S}=\oint \vec{E}.d\vec{l}\;(Jackson, 1962) \end{array}$$

describes how the variation of the flux of the magnetic field over time induces an electric field, where ***B*** is the magnetic field, ***E*** the electric field, ***l*** the contour and ***S*** the surface. The electric field creates a secondary electric current in a nearby conductor (such as brain tissue) in an opposite direction to the primary current. This secondary current shows the same symmetric distribution around the axis of the coil as the primary electric current (Tofts, 1990; Battocletti et al., 2000).

#### Electric and Magnetic Field Modeling

**FIGURE 2:**
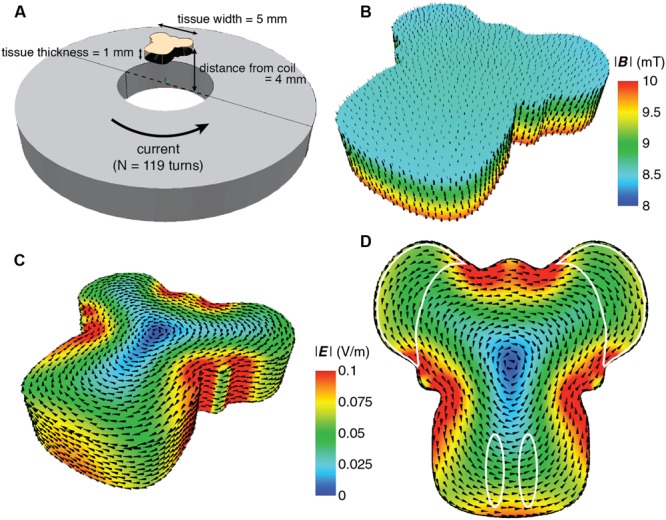
**Detailed finite element modeling of the magnetic and electric fields in the explant tissue. (A)** Schematic overview of the spatial relation between the coil and the explant. The coil has 119 turns with counter clockwise current flow. Coil dimensions are listed in **Table 1**. The overall dimension of the explant is 5 mm in width and length, and 1 mm thickness. It is placed 4 mm above the surface of the coil, centered along the coil axis. **(B)** Simulated magnetic field distribution in the explant. The arrows indicate the direction of the magnetic flux. For illustration purposes, the intensity scale has been limited to between 8 and 10 mT in order to show the intensity gradient throughout the explant. **(C)** Simulated electric field distribution in the explant. The arrows indicate the direction of the induced current flow. For this figure the upper scale has been limited to 0.1 Vm^-1^ to demonstrate the range of *E* intensity within the explant, as it is not visible in **Figure 1E**. **(D)** Electric field distribution looking at the side of the tissue closest to the coil. The areas of interest are outlined in white and all receive the same *E* intensity.

Based on the Maxwell–Faraday equation, the amplitude of the induced electric field depends on magnetic field amplitude, how fast it changes over time and its direction (axial components). To simplify our simulations and increase reproducibility, we chose to build a round coil: along the axis of the coil, the magnetic field is maximal, unidirectional, and parallel to the coil axis. In order to induce such a unidirectional magnetic field at the target, the inner coil diameter had to be larger than the dimensions of the explant. Thus for our particular setup, we made the inner diameter 10 mm so that the whole explant lay within a unidirectional homogenous magnetic field (**Figures 1B,D** and **2A**). Also, for reproducibility of positioning we made the outer coil diameter the same as the culture well (30 mm; see coil design, below). To estimate magnetic field homogeneity, we calculated the influence of its different axial components. In an axisymmetric plane, the vectorial magnetic field 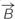 is composed of the sum of a vertical field component B_z_ and a horizontal component B_r_, with their directional unit vectors 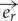 and 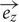, and can be written as: equation

(2) $$\begin{array}{c} \vec{B}=B_{r}\vec{e_{r}}+B_{z}\vec{e_{z}}\; \end{array}$$

Modeling of the predicted magnetic field (MATLAB, MathWorks, USA; Battocletti et al., 2000; Simpson et al., 2001) showed a homogenous field at the location of the explant (**Figures 1B** and **2B**), 4 mm vertically above the base of the coil (1 mm plastic shell + 2.5 mm of culture plate plastic and free distance + 0.5 mm half thickness of the sample). With a value of 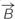 = 9.6 mT at the explant (**Figures 1B,C**), the vectorial component of the magnetic field 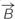 at a radius of up to 2.5 mm from the center of the coil was mainly vertical, B_z_ (9.6–9.27 mT), while the horizontal component B_r_ approached 0 (**Figures 1B,C**). Since the explant is only 5 mm long/wide (see Requirements for *In vitro* Magnetic Stimulation), even the outer components of the tissue, which are the regions of interest for our experiment, receive an effectively homogeneous unidirectional magnetic field. However, any movement of the target tissue along the vertical or horizontal axis of 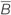 can modify the magnetic field intensity 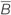 at the target location, depending on experimental requirements. We also modeled the induced electric field as the critical parameter in our setup (Yedlin et al., 1974; Chan and Nicholson, 1986; Rohan et al., 2014). The explant and the coil were modeled with the finite element package MagNet v 7.0 (Infolytica, Inc., Canada). Since the tissue consists of a mixture of neurons, glia, and axons that are orientated in longitudinal, transverse and oblique directions, the tissue was modeled by a homogeneous conducting volume with isotropic conductivity of 0.33 Sm^-1^, and electric permittivity and magnetic permeability of free space (**Figure 2A**). The magnetic and electric fields were computed using the 3-D time-harmonics solver via the T-Ω method, and peak field strengths were subsequently scaled by the maximum rate of change of the current (d*I*/d*t* = 20 kA s^-1^; Deng et al., 2011). As expected at the absolute center of the coil the induced electric field was zero and it increased to the coil edge. Because of the symmetric dimensions of the tissue and coils, the regions of interest for our study (the cerebellar lobe and inferior olivary nucleus) were equidistant from the coil’s center and thus electric fields of similar magnitude (∼0.05 Vm^-1^; **Figures 1E** and **2C,D**) were induced within them. This electric field is at least two orders of magnitude below the electric field amplitude reported for activation of cerebellar neurons (Chan and Nicholson, 1986). Although this intensity suggests stimulation that is subthreshold for neuronal firing, the placement of the conductive tissue within the magnetic field can itself modify the induced electric current. Based on the symmetry of a circular coil around its central axis, the induced electric field creates secondary current loops parallel to the current in the coil in any neural tissue within the field. However, if free looping of the secondary current is not possible due to presence of conductor boundaries it will produce an accumulation of free charge at the boundaries of the conductor (in this case the interface between the edge of the explant and the surrounding air – O_2_/CO_2_ mix), which will create a secondary potential (*phi* φ) working in the opposite direction to the induced electric current. Hence, to limit the effect of accumulation of free charges and consequent disruption to current flow, our explant tissue was positioned vertically above the center of the coil (**Figures 1B,D,E** and **2A**).

#### Magnetic Waveform Requirements

**FIGURE 3:**
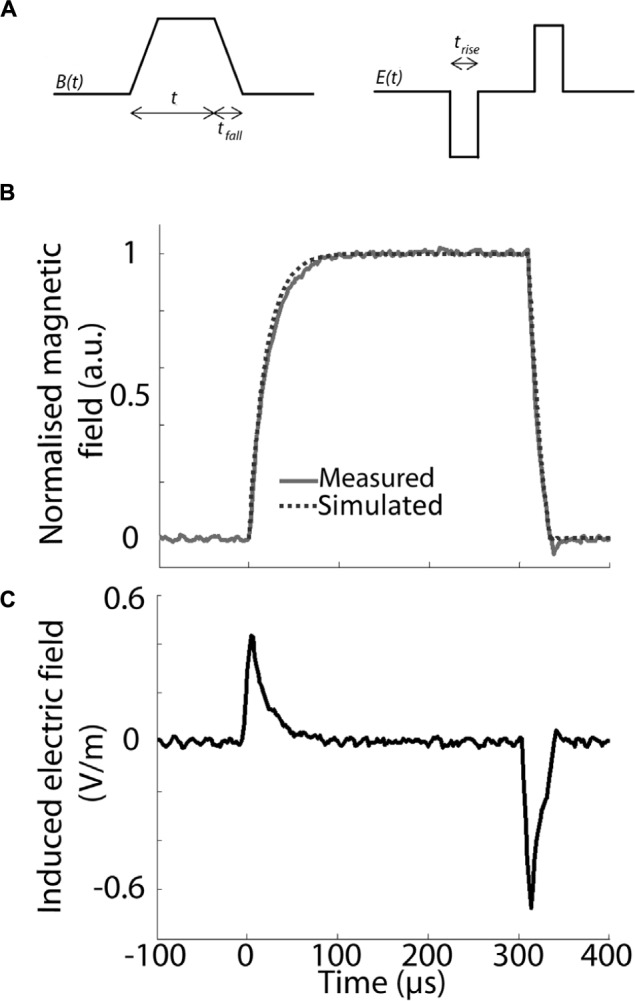
**Pulse waveform and parameters. (A)** Schematic representation of the trapezoidal waveform used to induce the magnetic field and the resulting predicted induced electric field inside a conductor (*E*(*t*)). Three different temporal domains are specified for this set-up: *t_rise_* = rise-time, *t_fall_* = fall-time, *t_1_* = pulse ON. *t_rise_* /*t_fall_* and *t_1_* are based on previous experiments and have a value of 100 and 300 μs, respectively. Note that there is a static magnetic field between rise and fall-times **(A,C)**, during which no current flows inside the target tissue. **(B)** Magnetic field intensity in normalized, arbitrary units (a.u.) induced inside the coil by a single pulse using the waveform shown in **(A)**. The intensity was modeled in TINA (dotted line) and measured via hall-effect (solid gray line), showing a tight correspondence between predicted and measured waveform. **(C)** Calculated, single pulse induced electric field in a round conductor at a radius of 2 mm from the central axis and 4 mm vertically above the top of the coil’s wiring.

The waveform of the magnetic pulse, specifically its rise- and fall-time, has a significant impact on the induced electric field in the target tissue. The induced secondary current is proportional to the derivative of the magnetic field in the target over time, so that the faster the rate of change of the magnetic field, the stronger the electric field induced within the target tissue. To have a constant induced electric field, the magnetic field needs to change at a constant rate over time with identical rise and fall-times to induce two electric field pulses of the same amplitude but with opposite directions (**Figures 3A,C**).

Based on previous *in vivo* parameters (Rodger et al., 2012; Makowiecki et al., 2014), we aimed to obtain a maximal magnetic field strength of 10 mT at the target tissue, with a rise-time of less than 100 μs and pulse length of 300 μs (Grehl et al., 2015). Because pulse shape alters the efficiency of neuromodulation (Goetz et al., 2016), we used a symmetric trapezoidal pulse with the same rate of current rise-and fall, in keeping with LFMS in humans (Rohan et al., 2014). Hall device (ss94a2d; Honeywell, USA) measurements confirmed the predicted magnetic field strength and pulse waveform (**Figure 3B**) at 4 mm above the base of the coil showing a tight correspondence of modeled (TINA, Texas Instruments, USA) and measured (Hall effect) pulse shape. This confirms a rise-time of <100 μs with a similar fall-time after 300 μs. For a peak current of 2 A, the maximum rate of change of the current is d*I*/dt = 2 A/100 μs = 20 kA/s. All coils were systematically tested and showed stability of magnetic field production for simultaneous and multiple activation (two at a time) at different stimulation frequencies.

### Generation of the Magnetic Field: Coil Construction and Circuit Design

To produce such a magnetic field, an inductor (coil) and electric circuit are required. The simplest appropriate model comprises a resistor-inductor (RL) circuit, in which the properties of one will alter the outcome of the other; thus these two components were designed in parallel.

#### Circuit Design and Construction

The circuit was created to generate parameters defined by previous experiments (300 μs pulses delivering 10 mT at a range of different frequencies), specifically the fast rise-time of the magnetic field (100 μs; Rodger et al., 2012), as well as to fulfill *in vitro* requirements (no excess heating or vibration). We also wanted the device to be able to generate electric field pulses repeated within a broad range of defined frequencies. Thus, we built the circuit so that the *minimum* possible pulse width was 200 μs, giving a maximal stimulation frequency of 5 kHz (1/200 μs).

**FIGURE 4:**
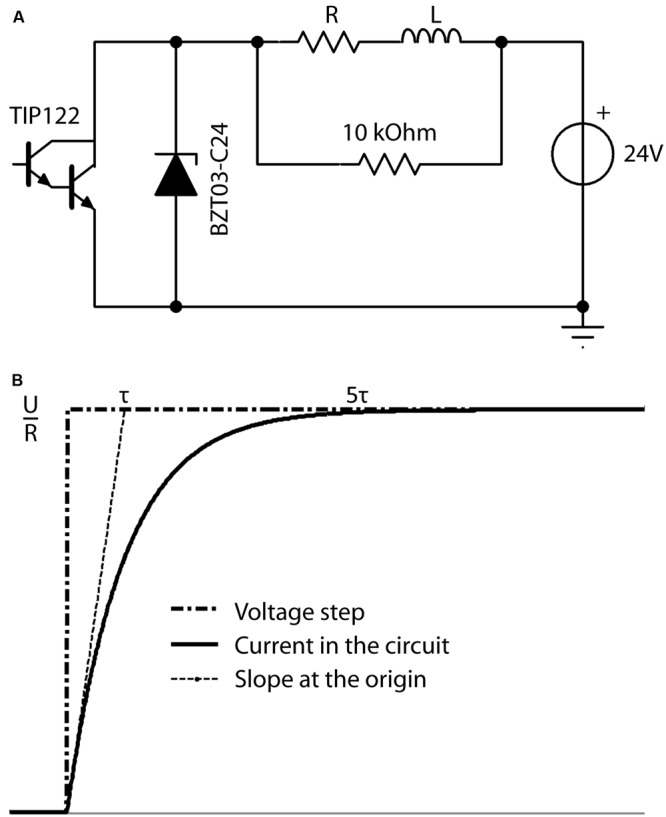
**(A)** Schematic overview of the electronic circuit used to produce the desired current in the coil (L). The microcontroller produces a squared waveform, which triggers the darlington transistor (TIP122) on and off to permit the 24 V power source to drive current through the RL circuit at the desired frequency during the desired period. The zener diode (BZT03-C24) is unidirectional and thus not involved in the active circuit. However, when the transistor is off, it is the energy stored in the coil L, which drives current through the R-BZT03 diode circuit. Thus when the transistor opens, the unidirectional BZT03-C24 diode is the primary limiter of the current level induced by the coils energy to control the fall time. **(B)** Definition of the characteristic time Tau (τ). Current intensity inside the circuit in response to increasing voltage steps reaches more than 99.9% of its maximum (i.e., *t_rise_*) after a time of ∼5*τ*.

The performance of the resistor-inductor circuit (RL circuit, **Figure 4A**) was simulated in TINA software. In an RL circuit, the response of the circuit to a voltage step is given by:

(3) $$\begin{array}{c} I(t)=\frac{U}{R}(1-e^{-\frac{t}{\tau }})\;\mathrm{with}\;\tau =\frac{L}{R} \end{array}$$

where *I*(*t*) is the electric current flowing in the circuit at a time *t, U* the value of the voltage step at time *t* = 0, *R* the total resistance of the circuit, and *τ* is the characteristic time for current to rise within the coil, a factor which depends on the inductance *L* of the coil and total *R* of the circuit. The two parameters *L* and *R* can be chosen as

(4) $$\begin{array}{c} t_{\mathrm{rise}}=5\tau =5\frac{L}{R} \end{array}$$

where the field intensity inside the circuit reaches more than 99.9% of its maximum after a time of ∼5τ (**Figure 4B**). The desired rise time (5τ) is defined as the time needed to reach the maximum magnetic field inside the coil and was defined as 100 μs in our system according to previous experiments (Rodger et al., 2012). However, the rise-time (*t*_rise_) is adaptable by changing the inductance *L* (e.g., changing the number of turns in the coil winding) and resistor *R*, while keeping *τ* constant.

During the fall-time, the energy stored inside the coil during the rise-time induces a current that powers the circuit through the zener diode. In order to have the same circuit response during this step, i.e., the same fall-time as the initial rise-time, a zener diode was used to limit the voltage generated by the coil to that of the power supply. Increasing the threshold (zener voltage) of the diode allows the stored energy to dissipate faster, leading to an increase in the rate of change of the magnetic field during the fall-time and thus regulating the pulse waveform.

To be able to systematically assess the effects of different magnetic stimulation frequencies, activation of the circuit needs to be easily adjustable to define the inter-pulse interval. This was achieved by using a programmable microcontroller card (Max 32, Chipkit), which could be programmed (C-based code) via USB connection to a standard PC, to select the stimulation duration, pulse length, and pulse spacing (frequency). In addition, the time of day and immediate or next day start of stimulation could be programmed with real-time, remote control and feedback options. In order to optimize efficiency of the experimental protocol, the circuit was designed to connect via 3.5 mm jacks to a total of 16 coils, with two coils being activated with same stimulation parameters at any one time; thus allowing up to eight different coil protocols to be programmed at a given time. In addition, each coil is driven by a separate circuit and we verified experimentally that their performance does not influence each other.

#### Coil Calculation and Construction

At the same time, the circuit inductor (coil) had to fulfill the requirements for pulse rise-time and magnetic field intensity and homogeneity, while also conforming to the needs of the *in vitro* culture system (e.g., sterility and stable temperature). To find a feasible solution, the magnetic field and the coil characteristic for a given geometry and a given current were modeled in MATLAB.

**FIGURE 5:**
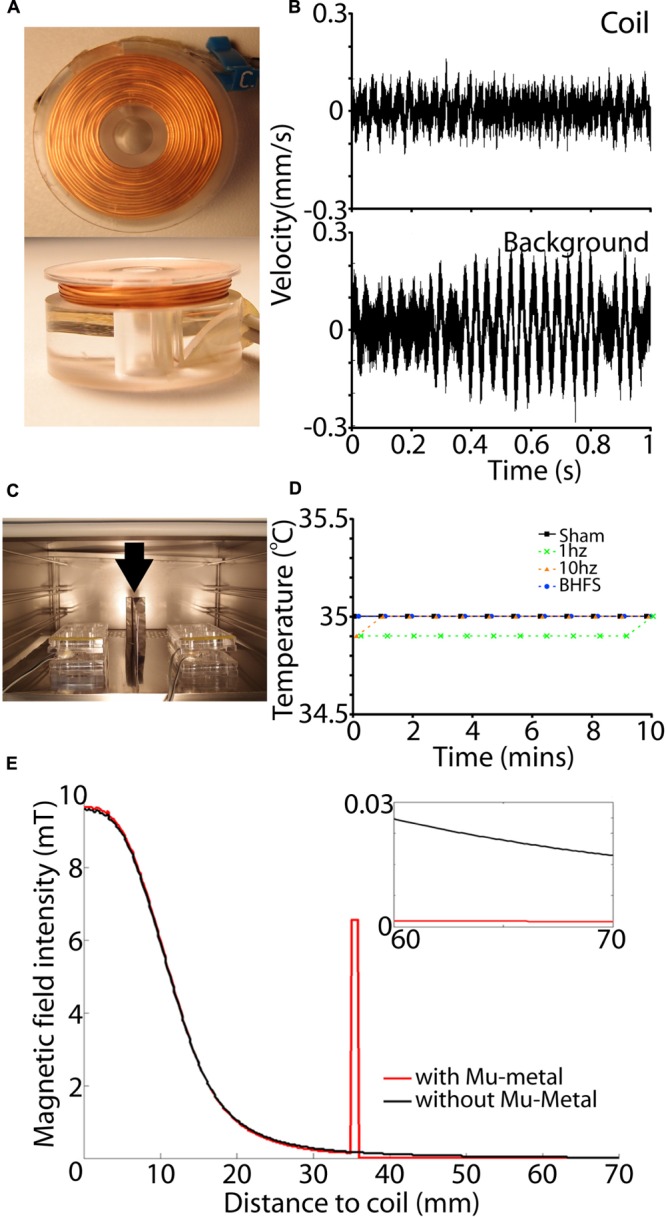
**Coil set-up and magnetic shielding. (A)** View of a single coil from the top (top panel) and side (bottom panel). **(B)** Vibration measurement of the coil (top) and background surface (below) in mm/s as measured by a single point vibrometer with OFV-534 compact sensor head for high optical sensitivity. Vibration amplitude of the coil is within background vibration. **(C)** View of the coil set-up within the incubator. A sheet of Mu-metal (min 1 mm thickness) is placed between adjacent cultures (vertical arrow). Also two wires can be seen passing to each six-well plate. This is because each coil is driven by a separate RL circuit. **(D)** Measurements of coil temperature during the 10 min stimulation period. For all frequencies and sham (unactivated coils) there was no temperature rise above that of the incubator (35°C). **(E)** Effect of Mu-metal on the magnetic field. Black lines indicate a produced magnetic field without Mu-metal shielding at a horizontal distance from the center of the active coil. Red line shows the magnetic field in correspondence with a Mu-metal shielding at 35 mm from the coil. Magnetic field intensity is concentrated by Mu-metal, leading to complete shielding with no detectable magnetic field at adjacent cultures beyond that distance (small inset).

First, to avoid perturbation of the culture environment, coil temperature (heating/dissipation) must not exceed the temperature of the incubator (35°C). Heat production (Joule effects) in the coil increases with increasing wire resistance and electric current intensity, which in turn is defined by the intensity required to produce the desired vertical magnetic field at the center of the coil. To avoid the costs and safety issues associated with using high voltages, we chose a standard 24 V power supply. Therefore, to balance between the required current intensity (2 A) and low internal resistance (large wire diameter) to minimize heating, we chose a wire of 0.4 mm diameter. Coils were wound manually using a custom built winder and chosen parameters are shown in **Table 1**. The base of the coil was made of *Poly methyl methacrylate* (PMMA), a non-conductive and non-magnetic polymer (**Figure 5A**). We verified the temperature stability of these coils with a K-type thermocouple (-40 to 260°C, Dick Smith Electronics Q1437, Australia) sensor attached to the top of the PMMA support with electrical tape; i.e., 1 mm above the top of the wire coil. We noted that the temperature never exceeded the incubator environment of 35°C (**Figure 5D**) thus ensuring that stimulation would not produce a thermal confounder within the tissue, which lies 4 mm from the coil. We also measured vibration (single-point vibrometer, Polytec, USA) and observed it was not above background of the bench surface (**Figure 5B**).

Second, to ensure the best balance between induction efficiency while avoiding magnetic field spillover, and therefore induction of eddy currents in other explants within the same culture plate, coil size should not exceed the culture well dimensions, i.e., 30 mm outer diameter. To optimize uniformity of the magnetic field at the tissue (vertical vector component) the inner diameter was set at 10 mm (**Figure 5A**). Also, we calculated in MATLAB and FEMM (Finite element method open-source software, USA) the minimal distance necessary to stimulate different culture wells simultaneously without interference between adjacent magnetic fields. Results show that at 85 mm distance between the two target tissues the influence of the adjacent magnetic fields was negligible (<11 μT) being less than the earth’s magnetic field (25–65 μT; Hulot et al., 2010). Hence, two explants cultured in diametrically opposite wells within one plate, could be stimulated simultaneously to increase throughput of different stimulation parameters (**Figure 1A**).

#### Isolation of Magnetic Stimulation Fields

To further increase the use of available incubator space (**Figure 5C**), we tested the feasibility of using Mu-metal (Magnetic Shields Limited, Tonbridge, UK) to shield adjacent culture plates from each other. FEMM was used to determine the required height and thickness of Mu-metal shields. Results showed that adequate Mu-metal shielding required a minimal sheet thickness of 1 mm, with a height of 100 mm to adequately shield adjacent culture plates separated by only 35 mm (**Figure 5E**). To prevent magnetic field interference in the vertical axis, each shelf within the incubator was fully covered with sheets of Mu-metal. Moreover, there was no direct contact between coils and Mu-metal to ensure that magnetic field strength was not attenuated by mu-metal interference.

### Biological Validation: Cellular Activation and Gene Expression Changes

In order to test whether our coil actually stimulated the tissue, we evaluated cellular activation and changes in gene expression following different stimulation parameters in our *in vitro* organotypic hindbrain explants, which contain the brainstem, cerebellum and their associated circuitry and develop as *in vivo* (Chedotal et al., 1997; Letellier et al., 2009). Animal housing and all procedures were performed under the guidelines established by *le comité national d′éthique pour les sciences de la vie et de la santé* in accordance with the European Communities Council Directive (2010/63/EU) and approved by the French Charles Darwin animal ethics committee (approval 01493.02). Hindbrain explants were cultured from Swiss mice at embryonic day 15 (E15) as previously described (Chedotal et al., 1997; Letellier et al., 2009). Briefly, embryo brains were quickly dissected in ice-cold Gey’s balanced salt solution (Eurobio) containing 5 mg/mL glucose. The hindbrain was isolated and the meninges removed. The right and left cerebellar plates were separated at the midline and the explants transferred onto Millicell membranes (pore size 0.4 μm) and cultured with medium containing 50% basal medium with Earle’s salts (Gibco), 2.5% Hank’s Balance Salt Solution (Gibco), 25% horse serum (Gibco), 1 mM L-glutamine (Gibco), and 5 mg/mL glucose. The culture day was designated 1 day *in vitro* (DIV). The medium was replaced every 2–3 days. Since our previous studies suggest that low intensity magnetic stimulation has less effect on normal vs. abnormal neural circuits (Rodger et al., 2012; Sykes et al., 2013; Makowiecki et al., 2014), we lesioned our explants to create a denervation/reinnervation model. Cerebellar plates were separated from their explant brainstem at *DIV 23* (equivalent to P17), placed adjacent to intact cerebellar tissue of a second explant (co-culture; **Figures 1A,D,E** and **2D**) and stimulated to induce reinnervation (Morellini et al., 2015).

Stimulation with 300 μs trapezoid magnetic pulses was delivered at frequencies of 1 Hz, 10 Hz or biomimetic high frequency stimulation (BHFS: 62.6 ms trains of 20 pulses, repeated at 6.55 Hz for 1 min, followed by 9.75 Hz for 8 min and then 6.15 Hz for 1 min; Rodger et al., 2012; Makowiecki et al., 2014; Grehl et al., 2015). The BHFS pattern was designed on electro-biomimetic principles to replicate endogenous patterns of electrical fields around activated nerves during exercise (Martiny et al., 2010; Rodger et al., 2012), and is based on the patent PCT/AU2007/000454 (Global Energy Medicine). These parameters delivered the number of magnetic pulses described in **Table 2**. LI-rMS was delivered through coils placed below individual culture wells (**Figures 1A** and **5A,C**) and different culture plates were separated by mu-metal (see Requirements for *In vitro* Magnetic Stimulation and Coil Calculation and Construction). Explants were stimulated for 10 min/day for 14 consecutive days, with the set-up only being disturbed to change the culture media. Because active coils generated neither heat nor vibration (**Figures 5B,D**), we used non-activated coils as stimulation controls (sham) for all experiments.

**Table 2:** Number of pulses delivered by LI-rMS.

	Single 10 m session	14 daily sessions
1 Hz	600	8 400
10 Hz	6 000	84 000
BHFS	108 840	1 523 760

To ensure that any biological effects induced by LI-rMS were consistent across different litters, 3 litters (32 embryos) were used to generate 16 co-cultured explants (cerebellum from one grafted onto a different explant as host) for each stimulation batch (see Circuit Design and Construction). Within these batches four co-cultures were stimulated for each frequency/sham. Thus each litter contributed to every experimental group, and each group included explants from different litters.

#### LI-rTMS Activates Cerebellar Neurons

Cellular activation by LI-rMS was evaluated by immunohistochemistry for c-fos after a single 10 min stimulation session, delivered 72 h after denervation to avoid observing acute effects of the lesion. Four hours after stimulation explants were fixed with 4% paraformaldehyde for 4 h at 4°C, rinsed 3 min × 5 min in phosphate buffered saline (PBS) containing 0.25% TritonX (PBS-T) and blocked in 20% donkey serum for 2 h at RT prior to incubation overnight at 4°C in primary antibody diluted in PBS-TG (PBS-T containing 0.2% gelatine and 0.018 g/ml Lysine). Primary antibodies were rabbit anti c-fos (Santa Cruz, 1:750) plus one of four different antibodies to label specific cell populations (Celio, 1990; Weyer and Schilling, 2003): Purkinje cells – mouse anti-CaBP-28k (1:2000; Swant), GABAergic interneurons – goat anti-Parvalbumin (PV, 1:3000; Swant), granule cells – mouse anti-NeuN (1:200; Millipore), astrocytes – mouse anti-GFAP (1:500; Sigma). The next day, explants were washed 3 min × 5 min in PBS-T and labeling was visualized with Cy3-conjugated donkey anti-rabbit, AF488-conjugated donkey anti-mouse or AMCA-conjugated donkey anti-goat (all 1:200 in PBS-TG; Jackson Laboratories) for 2 h at RT. Finally, explants were rinsed and mounted in Mowiol, then analyzed using epifluorescence microscopy (DM 6000B; Leica). As c-fos labeling appeared to be evenly distributed throughout the cerebellar tissue, three sites in each co-cultured cerebellar plate were selected semi-randomly for quantitative analysis. The number of c-fos positive cells was counted within the image z-stacks and expressed per unit area. After verifying homogeneity and normality of the data, group averages were compared by ANOVA and Tukey *post hoc* analysis.

**FIGURE 6:**
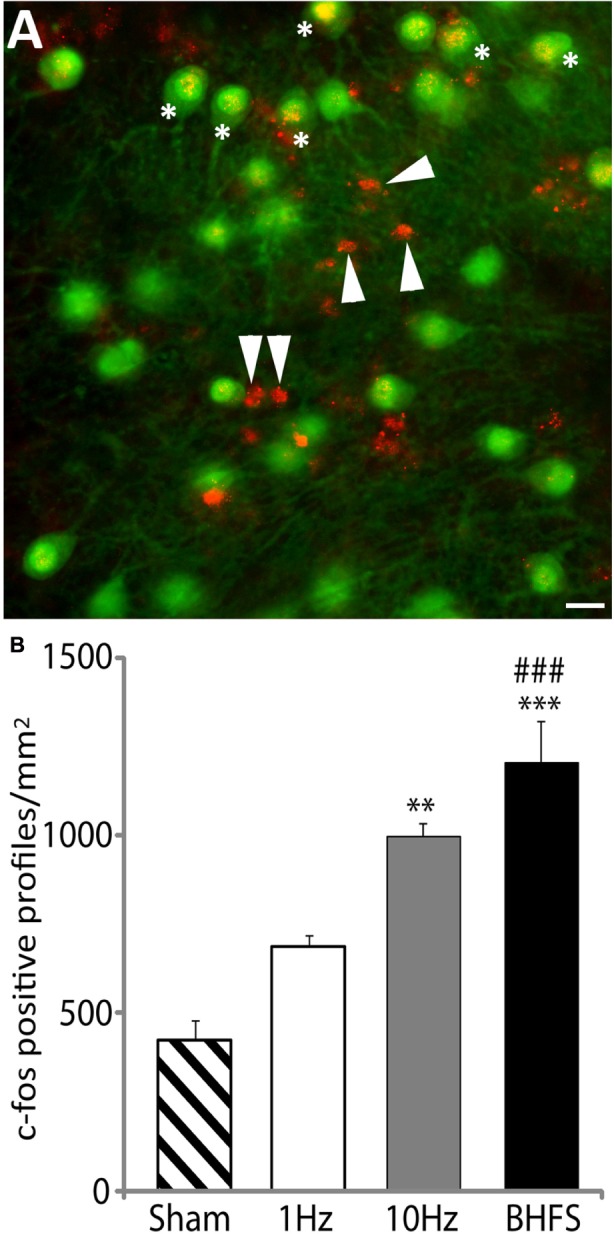
**c-fos labeling following LI-rMS in the cerebellar plates. (A)** An example of c-fos labeling after stimulation by BHFS. In this case c-fos (red) is co-labeled with calbindin (green) to reveal Purkinje neurons. C-fos labeling is located in some of the Purkinje cell nuclei (^∗^) and in other calbindin-negative profiles (arrowheads), which are the size and location of either granule or stellate neurons. Bar = 25 μm. **(B)** Histograms show the number of c-fos positive cellular profiles per mm^2^ in sham (unstimulated) controls, and explants stimulated with 1 Hz, 10 Hz or BHFS (*n* = 4 for each group; all four groups contain explants from the same 3 litters). Error bars are standard error of the mean. Cellular labeling in comparison to sham: ^∗∗∗^*p* < 0.0001; ^∗∗^*p* < 0.01. 1 Hz vs. BHFS between group comparison: ^###^*p* < 0.001 (ANOVA followed by *post hoc* Tukey pairwise comparisons).

The number of c-fos positive profiles (**Figure 6A**) significantly increased following LI-rMS at high frequency stimulation (10 Hz and BHFS) in comparison to sham (*F*_3,15_ = 20.83, *p* = 0.000; *p* = 0.002; and *p* = 0.000 respectively, **Figure 6B**). In contrast LI-rMS at 1 Hz induced only an intermediate increase in c-fos labeled cells, which was not different from either sham (*p* = 0.108) or 10 Hz (*p* = 0.125), but was significantly less than BHFS (*p* = 0.001). Qualitative analysis of double-labeled explants revealed that c-fos only co-localized with neuronal markers (calbindin, parvalbumin, and NeuN) but not with astrocytes (GFAP), suggesting that a single session of sub-threshold rMS activates cerebellar neurons.

#### LI-rMS Modifies Expression of Plasticity Related Genes

Low intensity repetitive magnetic stimulation induced changes in gene expression were assessed by RT-qPCR of RNA extracted from either the cerebellar plate or the inferior olivary region of lesion-stimulated/sham explants 24 h after the last stimulation (*DIV* 43). Tissue from five cerebella plates and inferior olive regions were pooled and total RNA was extracted using Trizol (Life Technologies) according to manufacturer’s instructions (Chomczynski and Sacchi, 1987) and stored at -80°C. RNA concentration and purity was measured by the ratio of absorbance at 260/280 nm and only those samples with a ratio 1.8–2.1 were kept. 200 ng of total RNA was reverse transcribed in a 20 μl reaction using a High Capacity cDNA Reverse Transcription Kit (Applied Biosystems). cDNA was amplified on a LightCycler^®^ 480 (Roche Applied Bioscience, USA) for 10 μl reaction volume using SYBR Green I Master Mix (annealing temperature 58°C, 50 cycles). Housekeeper primers were obtained from the mouse- reference gene panel (Tataa Biocenter, Sweden) for *hypoxanthine phosphoribosyltransferase 1* (HPRT 1). Primer sequences of test genes (TM = 59.0–59.6) were designed as follows:

   BDNF: forward TCACTGGCTGACACTTTTGAGCA,
   reverse CGCCGAACCCTCATAGACATGTTT.
   Pax3: forward AGCAAACCCAAGCAGGTGACA,
   reverse AGGATGCGGCTGATAGAACTCACT;
   Sia2: forward AGCACAATGAACGTGTCCCAGAA,
   reverse GAGCCAGGTTGCACCTTATGACA;
   Sia4: forward TTCCGGCATTCTGCTAGACAGTG,
   reverse CGAAAGCCTCCAAATGCTCTTTGC.

Raw data were pre-processed with Lightcycler 480 software (Roche Applied Bioscience, USA) and only samples with >90% efficiency were retained for analysis. Gene expression was normalized to housekeeper gene expression. All samples were amplified in triplicate and the mean used to calculate gene expression in each tissue sample. Normalized mean expression [log2(2-ΔCp); Livak and Schmittgen, 2001] was used to determine differentially expressed genes between each LI-rMS group. Normality and homogeneity of data were verified and intergroup comparisons were made by ANOVA and *post hoc* Tukey pairwise comparisons.

**FIGURE 7:**
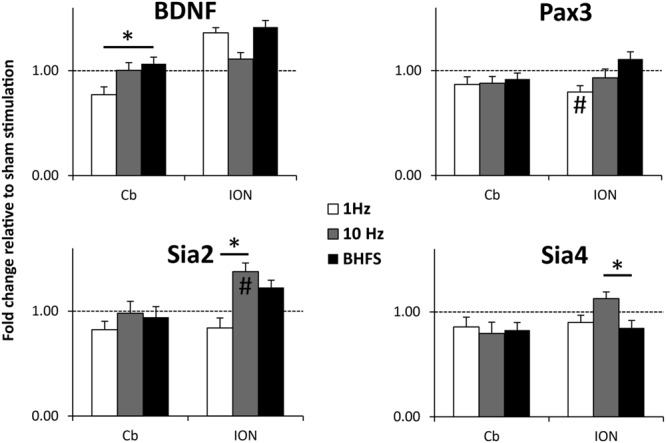
**BDNF, Pax3, Sia2, and Sia 4 mRNA expression levels in the cerebellar plate (Cb) and inferior olive (ION) normalized to sham (unstimulated) controls.** Explants were stimulated with 1 Hz, 10 Hz or BHFS (*n* = 5 for each group; all four groups contain pooled tissue from the same 15 litters). Histograms show mRNA levels as fold-change relative to sham (horizontal dotted line at threshold change of 1). Error bars are standard error of the mean, ^∗^ indicates *p* < 0.05 and **#** indicates significantly different compared to sham stimulated controls (*p* < 0.05) (ANOVA, *p* < 0.05, followed by *post hoc* Tukey pairwise comparisons).

We observed significant changes of gene expression following different stimulation protocols (**Figure 7**). In the denervated/reinnervated cerebellar plate, BDNF expression was significantly greater following LI-rMS at BHFS compared to 1 Hz (*p* = 0.025). In addition, we observed changes in expression of other genes expressed within our system, validating that our equipment has a biological effect on the explants. In the reinnervating ION, 1 Hz tended to reduce gene expression: Pax3 expression was reduced following 1 Hz compared to sham (*p* = 0.045) and Sia2 expression was reduced following 1 Hz compared to 10 Hz (*p* = 0.000) and BHFS (*p* = 0.004). In contrast 10 Hz increased expression of the Sia enzymes: Sia2 expression following 10 Hz was greater compared to sham (*p* = 0.01) and 1 Hz (*p* < 0.000), and Sia4 was increased following 10 Hz compared to BHFS (*p* = 0.035).

## Discussion

Here, we describe the construction of a magnetic stimulation device scaled-down for low intensity stimulation of *in vitro* neuronal preparations. The device can be tailored to meet the requirements of specific *in vitro* models: programming can define different stimulation frequency or duration; and plug-and-play features allow interchange of coils to modify wave-form and size of stimulation area, and power supply/resistors to alter stimulation intensity. We validate the device by showing frequency-specific effects of magnetic stimulation on neuronal activation and gene expression in an *in vitro* model of neural circuit repair. This simple device is therefore a valuable tool for characterizing the biological effects of magnetic stimulation, by delivering a range of defined low-intensity stimulation parameters. Such information may be useful to facilitate the optimisation of disease-specific non-invasive brain stimulation protocols in human patients.

### Concept of Tailoring the Device

The biological effects of magnetic stimulation remain poorly characterized because most studies have been undertaken in humans where the cellular and genetic changes cannot be adequately measured. While clinical high-intensity rTMS/TMS is thought to depolarise neurons and induce activity-dependent plasticity (Quentin et al., 2013; Volz et al., 2014), the equally striking neuromodulation elicited by lower intensity sub-threshold stimulation, e.g., diffuse LFMS/PMF or focal LI-rTMS, cannot involve these mechanisms but activate molecules and signaling pathways that are currently unknown. In order to optimize this stimulation paradigm, it is necessary to know what magnetic, and therefore electric, field is being applied to the tissue in order to characterize stimulation-specific effects.

The effects and mechanisms underlying low intensity magnetic stimulation are beginning to be investigated in animal and *in vitro* studies. Focally targeted LI-rTMS in animal models has required technical advances to generate new coils; such as implantable sub-millimeter-coils that activate local neurons and downstream circuits (Bonmassar et al., 2012; Park et al., 2013) and small non-invasive external coils for examining cell and circuit effects (Rodger et al., 2012; Makowiecki et al., 2014). Stimulation devices have also been created specifically for *in vitro* studies (Roth and Basser, 1990; Maccabee et al., 1993; Ahmed and Wieraszko, 2009, 2015; Basham et al., 2009; RamRakhyani et al., 2013). However, like human coils (Post et al., 1999; Rotem and Moses, 2008; Stock et al., 2012; Vlachos et al., 2012; Ma et al., 2013; Lenz et al., 2016) they cannot be applied for repeated stimulation sessions within an incubator and thus any observed effects of stimulation cannot be separated from environmental confounders. Importantly, it is known that changing the *in vitro* tissue environment can modulate the biological response to magnetic fields (RamRakhyani et al., 2013). Our solution for investigation of low intensity magnetic stimulation *in vitro* was to develop a small coil that can be used within an incubator to produce defined magnetic and electric fields at any given location within organotypic cultures. The advantages of this approach are that: (1) the homogeneity and symmetry of the magnetic field ensured that tissues lying on the same circumference from the coil axis, as for our tissue of interest, were exposed to electric fields of equivalent strength, which we show are biologically active; (2) the magnetic field created an induced current flow that is isoplanar with the tissue so its effect would be maximal (Wagner et al., 2009); (3) by placing the tissue within the axis of the coil we minimized boundary effects and optimized current circulation; and (4) the coils did not perturb the tissue culture environment, neither vibrating nor generating sound above background noise, or heat above the incubator temperature of 35°C, even with high frequency and prolonged stimulation protocols. While defined homogenous magnetic fields can be achieved by placing the target tissue within the axis of a Helmholtz coil solenoid (Montgomery, 1969) and the effects of different frequencies evaluated (Meyer et al., 2009), these coils are physically large and thus not well-adapted for use in the limited space of an incubator. Moreover, in contrast to Helmholtz solenoids, our small coils and use of mu-metal shields permitted multiple stimulation protocols to be tested simultaneously in a small space without fields from adjacent coils interacting.

In addition, the advantage of this apparatus is that its plug-and-play design permits changes to stimulation parameters are relatively easy and fast to accomplish, and do not require altering the basic design of the electronic circuit, thus increasing its applicability to different experimental requirements, e.g., organotypic culture of the cerebellum, cortico-striatal circuits or hippocampus, or microfluidics. It is relatively simple to design and build coils to deliver stimulation to any shape or size target, whether it be in culture or in animal models. As long as the stimulator/coil connection is not hard-wired (standard 3.5 mm jacks are used here) different coils can readily be exchanged (**Table 3**). Changes to the Zener diode and the power supply can further increase the flexibility of this system. In this way, it will be possible in future to compare *in vitro* the cellular and molecular changes induced by a range of stimulation parameters (e.g., pulse width, pattern, and waveform) used in a range of low intensity magnetic stimulation studies in humans (Capone et al., 2009; Martiny et al., 2010; Di Lazzaro et al., 2013; Rohan et al., 2014). In addition, to increase reproducibility and applicability, this device was designed to be as cost-efficient and mobile as possible, by operating automatically (no necessity to be connected to a dedicated computer once the microcontroller card is programmed) and without the requirement of amplifier set-ups and waveform generators.

**Table 3:** Parameters (coil and circuit power) that have been used in the same or similar LR circuits to deliver defined magnetic fields in different experimental contexts.

	*In vitro*		*In vivo*		
	Organotypic	1° culture^1^	SC^2^	Vis Cx^3^	Mot Cx^4^
Circuit power (V)	24	12	9	9	100
Magnetic waveform (μs):					
Pulse length	300	300	200	220	400
Rise time	100	300	100	220	400
Coil diameter (mm):					
Outer	30	16.2	8	8	8
Inner	10	8	4	6	6
Height	3.5	10	6	6	7
Cu wire:					
Diameter (mm)	0.4	0.25	0.25	0.25	0.125
Number of turns	119	462	300	300	780
Induced magnetic field (mT)	10	13	10	12	119

*1°, primary neuronal culture; MotCx, motor cortex; SC, superior colliculus; VisCx, visual cortex. ^1^Grehl et al., 2015; ^2^Rodger et al., 2012; ^3^Makowiecki et al., 2014; ^4^Tang et al., 2015.*

### Biological Validation: Frequency Related Effects of LI-rMS on Cell Activation and Gene Expression

To validate our device, we examined cellular activation and gene expression in a cerebellar model of denervation and reinnervation. High intensity rTMS can activate cerebellar circuitry in human subjects (Koch, 2010) and increase expression of the immediate early gene c-fos in hippocampal organotypic slice cultures in an activity-dependent manner (Hausmann et al., 2001). We show for the first time that LI-rMS differentially upregulates c-fos in cerebellar neurons 4 h after the end of stimulation, according to the stimulation delivered. Given that the induced electric field strength of <0.1 Vm^-1^ is below action potential threshold, it is unlikely that the c-fos upregulation observed in our study reflects neuronal firing. Rather, c-fos upregulation may have been due to increases in intracellular calcium, which we have previously demonstrated in response to LI-rMS (Grehl et al., 2015), and which can upregulate c-fos expression (Gissel et al., 1997). This hypothesis is supported by the intermediate non-significant increase in both c-fos positive cells (this study) and intracellular calcium observed in cultured cortical neurons (Grehl et al., 2015) following 1 Hz stimulation.

We also investigated the effects of LI-rMS on the expression of four candidate genes, which are involved in olivo-cerebellar development and plasticity (Morrison and Mason, 1998; Sherrard and Bower, 2001; Avella et al., 2006; Bosman et al., 2006; Zhang et al., 2007; Sherrard et al., 2009; Sherrard et al., 2013). Although, it has been shown that magnetic stimulation alters gene expression in different neuronal populations *in vivo* and *in vitro* (high intensity: Funke and Benali, 2011; Stock et al., 2012; Vlachos et al., 2012; Ma et al., 2013, low intensity: Rodger et al., 2012; Makowiecki et al., 2014; Grehl et al., 2015) we show for the first time stimulation-related effects of LI-rMS on cerebellar and inferior olive tissue. Here, we show that LI-rMS induced stimulation-related changes in BDNF and Sialtransferase 2/4 expression. Similar to high intensity stimulation (Gersner et al., 2011; Wang et al., 2011), we show that BDNF expression is greater following high frequency compared to low frequency rMS (BHFS vs. 1 Hz). Our data also show for the first time that genes expressed in the olivocerebellar system (Maisonpierre et al., 1990; Avella et al., 2006; Schuller et al., 2006; Sherrard et al., 2013), are regulated by LI-rMS in a frequency specific manner. Pax3 and Sia2 are regulated in concert, which is consistent with their biological relationships: Pax3 induces Sia2 expression (Mayanil et al., 2000) and both are less strongly expressed in the ION following 1 Hz stimulation. Although, as new findings, the expression changes to Pax3 and Sia2/4 *per se* cannot be considered as validation of our machine; they do validate that rMS delivered by our device induced a biological effect in the absence of confounders such as perturbation to pH, temperature, or vibration. Future studies will examine whether these changes in gene expression are accompanied by changes in reinnervation in the model.

Frequency specific gene regulation has been previously demonstrated in different *in vitro* models (Stock et al., 2012; Grehl et al., 2015) thus validating our device. However, although our data on BDNF expression are similar to results obtained in awake animals (Gersner et al., 2011) and *in vitro* (Wang et al., 2011) using suprathreshold stimulation via human coils, our data reveal for the first time that such changes are due to a specific magnetic stimulation protocol without cellular function confounders (e.g., possible stimulation-induced firing, animal/cellular stress, temperature, or pH change). The importance of excluding these confounders is highlighted by the diametrically opposing changes to BDNF expression depending on whether the animal undergoing rTMS was awake (increased) or anesthetized (decreased; Gersner et al., 2011). Thus our specialized *in vitro* LI-rMS equipment provides relevant insight into fundamental cellular mechanisms of low intensity magnetic stimulation over a very large range of stimuli (low to high frequency; 600–1.5 × 10^6^ pulses; **Table 2**). Importantly, this ability to differentiate primary effects (of the stimulation) and secondary outcomes (from altered neuronal activity or cellular stress for example) is crucial in order to develop new complex protocols that are appropriate for the treatment of human neuropathology.

## Conclusion

Custom made stimulation devices will help to systematically investigate and understand the processes underlying the many different effects of low intensity magnetic stimulation on biological tissue. Such understanding will help to guide optimization of therapeutic application and increase the possibility to custom-tailor magnetic stimulation in the clinical setting.

## Author Contributions

SG, DM, and RS designed the research; SG, DM, and CG undertook the work; SG, JR, and RS analyzed data, SG, DM, Z-DD, JR, and RS wrote the paper.

## Conflict of Interest Statement

Z-DD is an inventor on patents and patent applications related to TMS coil technology owned by Columbia University. All the other authors declare that the research was conducted in the absence of any commercial or financial relationships that could be construed as a potential conflict of interest.
